# A Disposable Passive Microfluidic Device for Cell Culturing

**DOI:** 10.3390/bios10030018

**Published:** 2020-02-29

**Authors:** Francesco Guzzi, Patrizio Candeloro, Maria Laura Coluccio, Costanza Maria Cristiani, Elvira Immacolata Parrotta, Luana Scaramuzzino, Stefania Scalise, Elisabetta Dattola, Maria Antonia D’Attimo, Giovanni Cuda, Ernesto Lamanna, Lucia Carmela Passacatini, Ennio Carbone, Ulrich Krühne, Enzo Di Fabrizio, Gerardo Perozziello

**Affiliations:** 1 Department of Experimental and Clinical Medicine, University of Catanzaro, Germaneto, 88100 Catanzaro, Italy; francescoguzzi@aol.it (F.G.); patrizio.candeloro@unicz.it (P.C.); coluccio@unicz.it (M.L.C.); costanza.cristiani@unicz.it (C.M.C.); parrotta@unicz.it (E.I.P.); scaramuzzino.luana@unicz.it (L.S.); stefania.scalise@unicz.it (S.S.); elisabettadattola@gmail.com (E.D.); mariaantonia.dattimo@libero.it (M.A.D.); cuda@unicz.it (G.C.); lamanna@unicz.it (E.L.); carmela.19@live.it (L.C.P.); ennio.carbone@ki.se (E.C.); 2 Department of Chemical and Biochemical Engineering, Technology University of Denmark, 2800 Kongens Lyngby, Denmark; ulkr@kt.dtu.dk; 3 Physical Sciences and Engineering, King Abdullah University of Science and Technology, Thuwal 23955-6900, Saudi Arabia; Enzo.DiFabrizio@KAUST.EDU.SA

**Keywords:** passive microfluidic device, cell culture, micro-bioreactor

## Abstract

In this work, a disposable passive microfluidic device for cell culturing that does not require any additional/external pressure sources is introduced. By regulating the height of fluidic columns and the aperture and closure of the source wells, the device can provide different media and/or drug flows, thereby allowing different flow patterns with respect to time. The device is made of two Polymethylmethacrylate (PMMA) layers fabricated by micro-milling and solvent assisted bonding and allows us to ensure a flow rate of 18.6 μL/h - 7%/day, due to a decrease of the fluid height while the liquid is driven from the reservoirs into the channels. Simulations and experiments were conducted to characterize flows and diffusion in the culture chamber. Melanoma tumor cells were used to test the device and carry out cell culturing experiments for 48 h. Moreover, HeLa, Jurkat, A549 and HEK293T cell lines were cultivated successfully inside the microfluidic device for 72 h.

## 1. Introduction

Traditional culture methods are expensive and require intense work. Microfluidic cell cultures provide low consumption of reagents and a low risk of contamination. A synoptic view of the advantages in microfluidic cell cultures can be found in the scientific literature [1]. Microfluidic systems provide an excellent control of microenvironments [2,3,4], which is useful for high-performance cellular screenings [5,6,7,8], due to a high level of functional elements [9,10,11] and sensor integration [12,13,14,15,16], and for generating different types of gradients [17,18,19,20]. Moreover, microfluidic platforms offer the possibility to dynamically and automatically modify the microenvironment in different ways [21,22,23] for several applications [24,25,26,27].

Most of the microfluidic devices used for cell culturing presented in the scientific literature are based on external pressure sources consisting of pumps or micro-pumps to ensure a constant flow rate [28,29,30,31], although pressure and electrical gradients are not the only ones to have been used [32].

In this work, we describe a passive Polymethylmethacrylate (PMMA) microfluidic device with several novelties in comparison to what can be found in literature (see Table S1 in Supplementary Materials for details). The layout ensures a homogeneous diffusion of the medium inside the culture chamber twice or three time faster than in other similar devices [33] (see Figure S1 in Supplementary Materials). This device could help the monitoring of cellular growth, while also minimizing the work of external operators. Nowadays, in fact, biologists have to constantly pay attention to the culture and they have to refresh the medium manually every day. This could be avoided with such a device, which also reduces the risk of contamination because there is no need to refresh or change medium during the entire experiment, thanks to the sizes and geometries of the wells that ensure a fluid supply is available for several days and help us to optimize the volumes in the reservoirs.

The presented device has the advantage to leave the culture chamber opened, unlike some work present in the literature [34], and it allows us to handle cells in a single culture chamber for a long period of time. This configuration allows us to easily inject the samples in the device and to recover them after the experiments for further studies or applications. This peculiarity allows us biologists and those who are not experts in microfluidics to use such devices. Moreover, flows inside the microfluidic chamber can be controlled and managed carefully, in order to optimize cell growth while carrying out screening protocols. We can operate this device for few days or a week using a large flow rate ranging from ca. 1 μL/h to ca. 18 μL/h, respectively considering the minimum and the maximum height possible for the fluidic column inside the reservoirs. Finally, the low cost of the used materials and fabrication processes (e.g., an order of magnitude lower than other devices fabricated in PDMS by soft-lithography) makes the device disposable. Approximately, the cost of our device would be around $5 and can be further reduced if mass fabrication technologies would be used, like injection molding.

The presented device lacks any external pressure sources. It is able to ensure a pressure gradient Δ*P*, thanks to the difference in height Δ*H* between the wells and the culture chamber, according to Stevino’s law:

Δ*P* = *ρ g* Δ*H* (1)

where *ρ* is the density of the fluid and *g* is the gravity acceleration. In scientific literature, it is possible to find other works about passive microfluidic devices [35,36,37,38]. The device we present has different advantages, such as a low-cost manufacturing process [39,40,41], the possibility to be placed into a traditional incubator using a Petri dish or into a specially dedicated mini-incubator, to control and monitor constantly physiological parameters, such as pH and dissolved oxygen and cellular growth by using sensors and an inverted optical microscope for 72 h. It has to be pointed out that, during design and manufacturing processes, it is possible to regulate the hydraulic resistances in such microfluidic devices by modifying the geometrical parameters of microchannels that connect the reservoirs to the culture chamber. In this way, it is possible to dispense different substances for the cells at different time instants, carrying out drug cellular screenings and/or stem cells differentiation protocols [42,43,44].

## 2. Materials and Methods

### 2.1. Materials

For the fabrication of this microfluidic device, two layers of PMMA produced by Röhm Italia SRL were used, assembled with solvent-assisted bonding by using absolute anhydrous ethanol produced by Sigma-Aldrich. The top and bottom layers were 6 mm thick and 3 mm thick, respectively. For the culture experiment, RPMI culture medium produced by Corning as well as 10% of FBS and 1% of streptomycin and penicillin were used. Melanoma tumor cells were used to test the device for 48 h. These cells are derived from primary culture and adapted to grow under artificial conditions, i.e., immortalized. Ethical Committees associated with “*Istituto Nazionale Tumori IRCCS-Fondazione Pascale*” of Naples granted ethical permission. Written informed consent was obtained from all patients in accordance with the Declaration of Helsinki to the use of human biological samples for research purposes. Moreover, to assess the validity of the proposed device as a general culture device, HeLa, Jurkat, A549 and HEK293T cell lines were cultivated for 72 h. Cells were purchased from ATCC.

### 2.2. Working Principles of the Device

**Figure 1:**
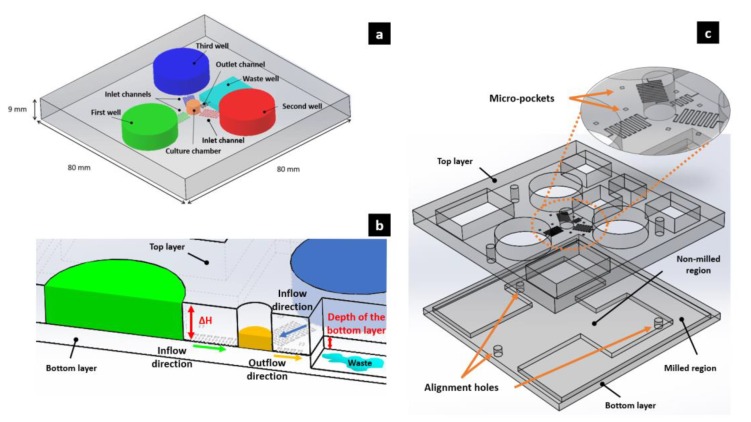
(**a**) A 3D schematic drawing of the assembled device. The bottom layer is not highlighted; (**b**) Section of the device. We can see the direction of the fluxes and the height ∆H of the fluids inside the reservoirs. The depth of the waste reservoir obtained manufacturing the bottom layer is visible. (**c**) View of the top and bottom layers with a zoom-in of the microchannels network. Three alignment holes help to fix the two layers with three screws during the bonding, while micro-pockets are useful to verify the presence of tilts or errors in the machined substrate with a profilometer. The milled region of the waste reservoir is located at a deeper level than that of the other tanks, in order to ensure a maximum pressure gradient between the waste reservoir and the culture chamber.

The microfluidic device presented in this work is completely passive. The microfluidic network and the three cylindrical reservoirs are manufactured onto the internal surface of the top layer. This surface is bonded with the non-manufactured internal surface of the bottom layer. A pressure gradient is generated by different liquid levels between the reservoirs and the culture chamber. The pressure gradient moves the liquids within the channels at a certain flow rate according to their fluidic resistance. Using this method, since the level of liquids changes while they flow from one reservoir to another, it becomes important to maintain the flow rate at as constant a rate as possible. To achieve this aim, we designed three cylindrical reservoirs (each one with a filling volume of ca. 2 mL), larger than higher, arranged at 120° around the central growth chamber (circa 60 μL), thus minimizing the decrease of height of the fluid in the reservoirs and the flow rate over time. In order to ensure there was a maximum pressure gradient between the culture chamber and the waste reservoir, the latter was manufactured onto the internal surface of the bottom layer, creating a tank (milled region in Figure 1c) located at a deeper level than that of the culture chamber and reservoirs. High attention must be paid to the tilt of the working plane, since a minimal inclination can also produce sensible pressure and flow rate variations.

Three inlet square channels connect the reservoirs to the culture chamber, and they have a pressure drop to their ends equal to:

∆*P* = *R* ∆*Q* (2)

where *Q* is the flow rate and *R* = 28.454 *ηL*/*a*^4^ is the Poiseuille’s resistance for a square channel of side *a* and length *L*, while *η* is the viscosity of the fluid [45] (see Table S2 in Supplementary Materials for details about the used values).

The device can ensure a flow rate ranging from circa 1 μL/h to circa 18 μL/h. These values were calculated respectively considering the minimum (*H_min, reservoirs_* = 3.1 *mm* and the maximum (*H_max, reservoirs_* = 6.0 *mm*) height possible for the fluidic columns inside the reservoirs. If there is no difference on heights between two columns of fluid, then there will be no gradient of pressure and, consequentially, no flow rate. It is simple to see in Equation (1), in fact, that when the height of liquid column into reservoirs is equal to the height of culture chamber (3 mm), there will be a ∆*H* = 0 and thus a ∆*P* = 0 and a ∆*Q* = 0. Pressure, flow rates and resistances values calculated can be found in Supplementary Materials (Equations (S1)–(S5)). An outlet channel connects the culture chamber to the waste reservoir (circa 6 mL). The pressure gradient allows a flow rate of 6.2 μL/h for each inlet channel (18.6 μL/h while considering the three inlet channels together), which decreases by circa 7%/day due to a decrease of Δ*H* while the liquid is driven from the reservoirs to the channels (Figure 1) (see Equations (S6)–(S11) in Supplementary Materials for the calculation details).

### 2.3. Flow and Pressure Drop Simulations

CFD simulations, by using COMSOL Multiphysics v5.2, were performed to assess the characteristics and the behavior of the microfluidic device. For such a purpose, streamlines, pressure drop as well as inlet and outlet velocity inside the microchannels were simulated. The simulations were performed considering a Newtonian fluid and no-slip conditions over all the walls of the microchannels. Meshes were controlled by physics and composed by tetrahedral elements, with a total of 6162 for the diffusion simulation and 92,744 for the pressure, velocity and streamlines simulations. According to the level of liquid in the reservoirs and to Equation (1), an inlet pressure of 58.932 Pa at the bottom of every reservoirs was set, considering a height of 6 mm (*H_max, reservoirs_*) and the values of *ρ* and *g* that can be found in Table S2 in Supplementary Materials. The outlet flow rate was set at the beginning of the outlet channel equal to 18.6 μL/h, the same of the total inlet flow rate, in accordance with the law of mass conservation.

Furthermore, the diffusion characteristics of the culture medium inside the central chamber was simulated. The initial concentration inside the culture chamber was set equal to 0 mol/m^3^ and a diffusion coefficient of glucose of D_c_ = 0.69∙10^−9^ m^2^/s was used [46]. The concentration of the diffusing chemical specie was supposed to be equal to 0.025 mg/mL, according to other works present in the literature [33]. Multiphysics coupling between Laminar Flow (spf) and Transport of Diluted Species (tds) physics was used. In any case, only the diffusion pattern of the solute inside the chamber coming from the three inlet channels was of interest, in order to ensure there was a homogeneous concentration of the growth medium that developed as fast as possible.

### 2.4. Device Manufacturing

PMMA layers were manufactured with micro-milling fabrication techniques using the machine model Mini-Mill/GX of Minitech Machinery Corporation. The spindle of the machine was controlled by Nakanishi E3000C, and produced by NSK Nakanishi. Cutting tools used for manufacturing processes were purchased from Performance Micro Tool, which have a declared diameter of 0.0040” (circa 0.100 mm) and 0.1181” (circa 3.0 mm). The microfluidic device has a square shape of 80 mm × 80 mm and a total thickness of 9 mm. First, PMMA layers were fixed on the working plane of the micro-mill and alignments along X-, Y- and *Z*-axes were performed. Since microchannels manufacturing is carried out with micro-milling techniques, there are some critical issues that can compromise manufacturing processes, such as the presence of tilted surfaces, incorrect alignment along axes, loss of alignment along axes due to software problems and/or the presence of eccentricity in the spindle rotation. All of these problems could affect the final product. The technical data of the device can be found in Table 1.

The two layers were put into an ultrasonic bath for 10 min. After that, they were placed inside an ethanol bath for 80 min and subsequently assembled with three screws placed inside the alignment holes. Finally, the system was transferred into a pre-heated pneumatic press, applying a temperature of 45 °C and a force of 1.5 kN for 70 min. As well as during micro-milling, thus solvent bonding has critical steps that must be considered. First, the presence of micro particles of dust or residues over the surfaces of interest could affect the process. Moreover, high pressure or temperature could occlude micro-channels or damage the device. On the other hand, low pressure or temperature could lead to non-bonded surfaces. Before assembling, some measurements were performed with the profilometer on seven micro-pockets with the same depth of the channels and a square shape of 0.6 mm × 0.6 mm. This allowed us to verify the presence of tilts or errors in the machined substrate.

### 2.5. Experimental Setup

**Figure 2:**
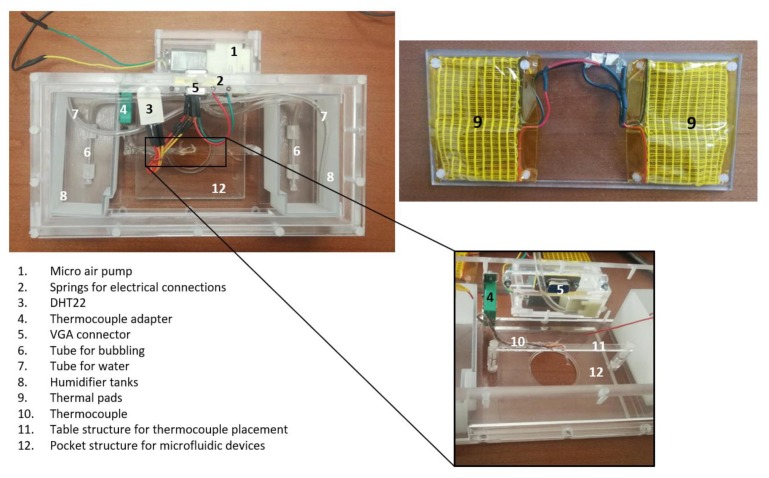
The mini-incubator used to characterize and validate the microfluidic device. Its dimensions make it possible to couple such a mini-incubator to an inverted microscope stage.

The microfluidic device was isolated as much as possible from the external environment to prevent evaporation and to keep a physiological environment for the cells. For such a purpose, a PMMA mini-incubator of 210.4 mm × 102 mm × 59 mm coupled to an inverted microscope stage was assembled (Figure 2). A CO_2_ source, a thermal-pad heated by an external electrical power source, a thermocouple, an environmental temperature and humidity sensors, a bubbling humidification system and specific holes allowing the insertion of tubes and wires were integrated inside the mini-incubator. Technical datasheets of each component used can be found in Tables S3–S6 and Figure S2 in the Supplementary Materials.

### 2.6. Device Experimental Characterization

To monitor the diffusion inside the culture chamber, food dyes were used. Generally red, blue and green produce the best contrasts in the visualizations [47]. We chose to use gel dyes and not powder to avoid precipitation. Reservoirs were filled with 1884 μL of fluid, and the culture chamber was filled with 59 μL, corresponding to a liquid height of 6 mm. Meanwhile, 200 μL were added to the waste reservoir after the priming of the microchannels to overcome hydrophobic forces. This experiment lasted 4 h, acquiring imaging with the optical inverted microscope in time-lapse from 7 different regions of interest inside the culture chamber every 10 min (see Figure S3 in Supplementary Materials).

### 2.7. Device Experimental Validation

Before performing the culture experiments, the device was sterilized for 90 min with an autoclave after being wrapped with aluminum sheets. Damages due to the overheating of the PMMA were not observed. First, melanoma tumor cells were used to test the device and carry out cell culturing experiment for 48 h. After checking the proper functioning of the device, HeLa, Jurkat, A549 and HEK293T cell lines were cultivated inside the microfluidic device for 72 h. For the culture experiments, the following priming protocol was carried out under the biological hood:

1. Priming of the channels with culture medium using suction tube under biological hood, thus preventing air bubbles;
2. Filling of the reservoirs with culture medium (circa 2 mL for each);
3. Suction of any volume accumulated in the culture chamber in the previous phases;
4. Loading of cells inside the culture chamber at the concentration of circa 62,500/mL. The culture chamber was filled with circa 59 μL of fluid containing circa 3700 cells;
5. Priming of the waste reservoir with 200 μL of culture medium to overcome hydrophobic forces.

The device was positioned in a Petri dish and placed inside a classical incubator for 48/72 h.

## 3. Results

### 3.1. Flow and Pressure Drop Simulations

**Figure 3:**
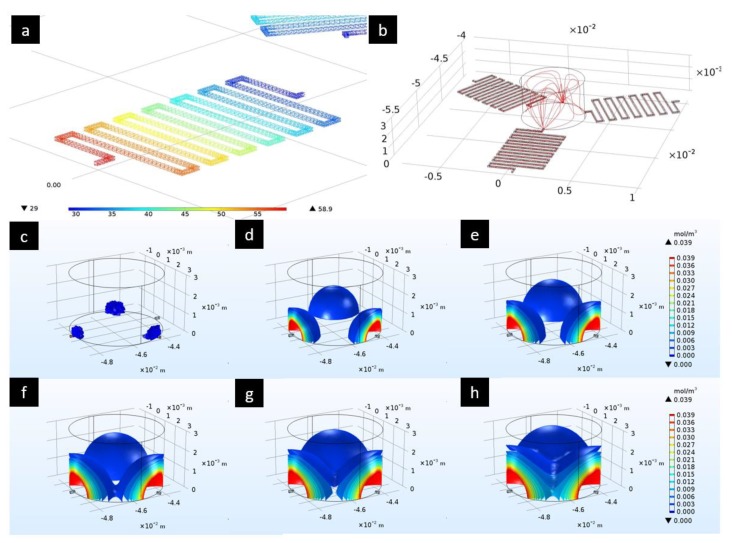
The top charts show the results obtained from the first simulations: (**a**) Pressure drop inside the microchannels [Pa]; (**b**) Streamlines of the laminar flow inside the microchannels and the culture chamber. Below are the results obtained from the diffusion simulation of the medium at different time instants [s]: (**c**) 0, (**d**) 300, (**e**) 500, (**f**) 650, (**g**) 800, (**h**) 1000. (For the results obtained in more time instances, see Figure S5 in Supplementary Materials).

In Figure 3, the results obtained after the simulations can be observed. A pressure drop of almost 30 Pa in the microchannels (Figure 3a) which leads to a pressure of circa 29 Pa at the bottom of the culture chamber can be noticed. This value is very close to 29.466 Pa (see Equation (S1) in Supplementary Materials). Streamlines (Figure 3b) are characteristic of a laminar flow, in agreement with the low Reynolds numbers present in microfluidics. Moreover, inlet and outlet velocities are very close to the average values calculated:

(3) $$\bar{v_{in}}=\frac{Q_{in,\;single\;channel}}{S}=\frac{0.001724219\;\frac{mm^{3}}{s}}{0.1\;mm*0.1\;mm}\approx 0.172\frac{mm}{s}\;$$

(4) $$\bar{v_{out}}=\frac{Q_{out}}{S}=\frac{0.005172658\;\frac{mm^{3}}{s}}{0.1\;mm*0.1\;mm}\approx 0.517\frac{mm}{s}$$

where *v* is the velocity, *Q* is the flow rate and *S* is the square shape area of the microchannels. Additional calculations can be found in Supplementary Materials (Equations (S1)–(S11)).

We reported the diffusion characteristics of the culture medium inside the central chamber (Figure 3c–h). We can note that after 1000 s the culture medium is homogeneously diffused inside the chamber thanks to the coupling with the laminar flow. A visualization of the real images acquired at circa 1000 s can be found in Figure S4 in Supplementary Materials.

### 3.2. Device Manufacturing

**Figure 4:**
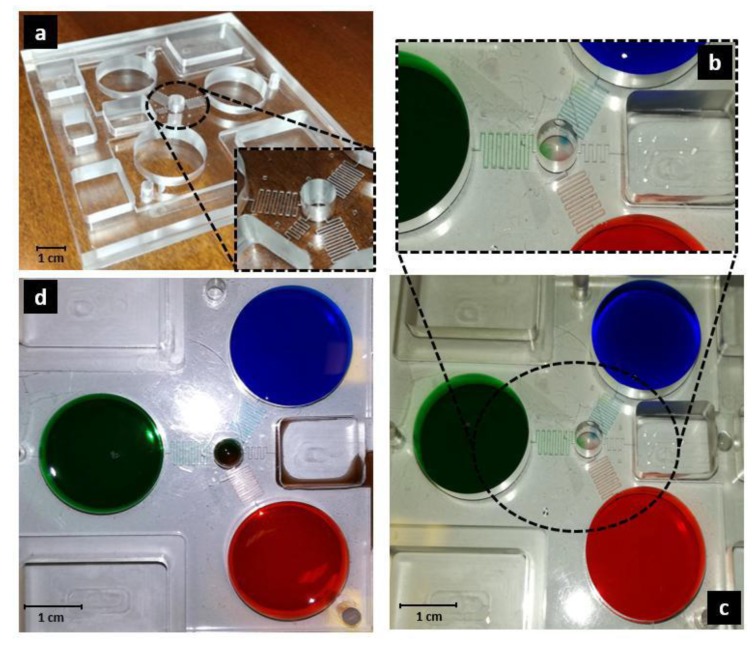
Isometric view of the fabricated microfluidic device. (**a**) A particular of the micro-channels is shown; (**b**,**c**) Diffusion after almost 600 s and (**d**) after 3 h during the experiment with food dyes, according to the diffusion simulation shown in Figure 3.

The microfluidic device is shown in Figure 4. Damage due to fabrication processes was not observed. Polymethylmethacrylate (PMMA) is a plastic material composed of methyl methacrylate polymers. It is known for its excellent properties of transparency to visible light and biocompatibility [48,49,50]. In fact, emerging biotechnology and biomedical research uses PMMA to create microfluidic lab-on-a-chip devices [51]. It is used in the fabrication of optical components, containers, pumps, filters, oxygenators and for applications with high demands for transparency. It is also widely used in orthopedic surgery as bone cement [52,53,54].

PMMA has a permeability to gas neglectable compared to other polymers usually used in microfluidic forms, i.e., PDMS [55]. The mean contact angle of conventional PMMA is almost 75°, this means that it is weakly hydrophilic [56]. As explained in Section 2.7, a priming of the waste reservoir with 200 μL of culture medium is preferred to overcome hydrophobic forces.

### 3.3. Device Experimental Characterization and Validation

The device was placed inside the mini-incubator for experimental validation.

During the diffusion experiment, a homogeneous mixing of the dyes inside the culture chamber was observed already after 3 h (Figure 4). Diffusion experiments were conducted inside the mini-incubator, where the temperature was set at 37 °C. Humidification was maintained between 80% and 90%.

The measured flow rate is circa 44.9 μL/h -17%/day (see Table S7 and Figure S6 in Supplementary Materials). The differences between theoretical and experimental values were mainly due to the real diameter of the tool used for the fabrication of microchannels, that involves a bigger width of the channels of circa 2% and, consequentially, a smaller hydraulic resistance and a bigger flowrate. Furthermore, the manufactured microchannels do not have a square shape, but instead a rectangular shape, and the new hydraulic resistance will be equal to *R* = 12 ηL/wa^3^ [45], where *w* is the bigger dimension (in this case the width) and *a* is the smaller dimension (in this case the height). Moreover, critical issues associated with micro-milling manufacturing processes and discussed in Section 2.4 must be taken into account.

After sterilization, immortalized melanoma tumor cells were used to test the device with a first cell culturing experiment for 48 h and the results can be found in Figure S9 in Supplementary Materials. HEK293T, A549, Jurkat and HeLa cell lines were used to carry out cell culturing experiments for 72 h. RPMI medium added with 10% of FBS and 1% of penicillin and streptomycin was used to feed the cells. Thanks to the optimizations of the volumes in the reservoirs, it was able to feed cells inside the central culture chamber for a longer time without having the need to re-fill the reservoirs with new medium for the entire experiment length of 48/72 h.

**Figure 5:**
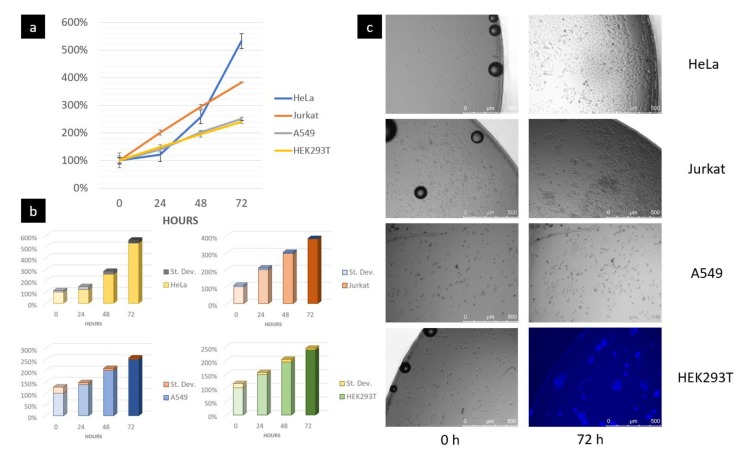
(**a**,**b**) Average percentage increase relative to 0 h and standard deviation evaluated on three different regions of interest for every cell line during the culture experiment in 72 h, considering an initial percentage of 100% at 0 h. Additional information about the positions of the regions of interest can be found in Figure S7 in Supplementary Materials Trends are comparable with results present in the literature [57,58,59,60]; (**c**) Growth of the cells in a specific region of interest. The presence of bubbles at time 0 h is because the photos have been acquired several minutes after cell seeding. For the HEK293T cell line, a DAPI staining was used. In fact, this cell line has a monolayer shape that makes the cell count more difficult without a fluorescent stain. For the images acquired at time instants 24 h and 48 h, see Figure S8 in Supplementary Materials.

Growth rates of cells obtained at the end of 72 h are comparable with results obtained into traditional Petri dishes (see Figure 5 and Figure S10 in Supplementary Materials) present in the literature [57,58,59,60]. Average percentage cellular growth was evaluated with *ImageJ* software on three different regions of interest. Additional information about the position of the regions of interest can be found in Figure S7 in Supplementary Materials. Adhesion on the PMMA substrate of the adherent cell lines (melanoma, HEK293T, A549, HeLa) was observed. Moreover, the combination of the used hydrodynamic parameters was able to not affect the proliferation of adherent and non-adherent cells (Jurkat). The viability of the cells was stated while considering their morphology and growth rate. Finally, acidification in the culture chamber was not observed, thanks to a continuous exchange of medium coming from the reservoirs. In fact, RPMI medium used has a pH ranging from 7.2 to 7.4 and contains Phenol red (also known as Phenolsulfonphthalein or PSP) a pH indicator frequently used in cell biology laboratories. Its color exhibits a gradual transition from yellow (*λ_max_* = *443 nm*) to red (*λ_max_* = *570 nm*), over the pH ranging from 6.8 to 8.2. Above pH 8.2 phenol red turns a bright pink (fuchsia) color [61,62]. In our experiment, no changes in colors were observed, i.e., no acidification of the medium.

## 4. Conclusions

In conclusion, we developed a passive microfluidic device allowing us to cultivate cells. According to the geometries used, the device ensures homogeneous concentrations of growth medium and/or drugs for 72 h, as predicted from the design and CFD simulations, and it can be used inside a traditional culture chamber or placed into a specific mini-incubator. The used fabrication process is low-cost and this makes the device disposable. The device is passive, so it does not need a complex experimental setup and specialized staff. Thanks to the optimizations of the volumes in the reservoirs, it was able to feed cells inside the central culture chamber for a longer time without having to re-fill the reservoirs with new medium for the entire experiments over 48/72 h.

The combination of the used hydrodynamic parameters allows us to generate a flow rate of a few μL/hour without affecting cellular adhesion and proliferation on the PMMA substrate. We observed a perfect adhesion on the substrate for the adherent cells and a proliferation comparable with results present in the literature for traditional Petri dishes [57,58,59,60]. Finally, no sign of acidification of the culture environment was observed thanks to the continuous exchange of the medium coming from reservoirs.

The opportunity to regulate hydraulic resistances makes it possible to implement a dispenser system of different substances for the cells at different times in microfluidic devices. We could also monitor physiological parameters of the cells by adding more sensors (pH, dissolved oxygen, etc.). We suppose that any further improvement could be relevant to using microfluidic devices for drug cellular screenings and differentiation of stem cells.

## Figures and Tables

**Table 1:** Technical data of the microfluidic device.

	Width (mm)	Height (mm)	Length (mm)	Diameter (mm)	Volume (μL)	Flow Rate (μL/h)
Total dimensions	80	9	80			
Inlet channel*	0.1	0.1	60			6.207 (−7%/day)
Outlet channel	0.1	0.1	20			18.61 (−7%/day)
Reservoirs		6		20	1884	
Culture chamber		3		5	58.875	
Reservoirs total filling volume					5652	
Predicted total flow rate						18.61 (−7%/day)

* Values which refer to a single inlet channel.

## Supplementary Materials

The following are available online at https://www.mdpi.com/2079-6374/10/3/18/s1, Table S1. Comparison between the presented device and some of continuous perfusion 2D cell culturing devices in literature. Table S2. Constants of interest used during the design of the microfluidic device. Table S3. Datasheet of Thermocouple RS Pro. Table S4. Datasheet of the SparkFun Electronics heating pad. Table S5. Datasheet of DHT22 temperature and humidity sensor. Table S6. Datasheet of Arduino Uno Controller. Table S7. Comparison between theoretical and experimental measured values of the device. Figure S1. Computational simulation evaluated over 4 h to assess the faster diffusion of substances in the presented device, comparing with some similar devices in literature [6]. The parameters used for simulation are shown below and are the same used by the authors of the cited article. Unlike their device, having among other things a smaller central culture chamber, a target concentration of 0.025 mg/mL is reached faster (1.5 h rather than 4 h) and increases over the time. Figure S2. Declared heating rate of the thermic pad used for our experiment and produced by SparkFun Electronics. Figure S3. Diffusion of the dyes over time during the experiment with food dyes. The experiment lasted 4 h, acquiring imaging with the optical inverted microscope in time-lapse from 7 different regions of interest inside the culture chamber. Figure S4. Diffusion of the dyes at ca. 1000 s. We can note how colors are starting to mix with each other, especially in the center of the culture chamber. Figure S5. Results obtained from the diffusion simulation of the medium at different time instants [s]: (a) 0, (b) 300, (c) 500, (d) 650, (e) 800, (f) 1000, (g) 1800, (h) 2400, (i) 3000, (l) 3600, (m) 4200, (n) 4800. Figure S6. Experimental trends of flow rate and pressure during a week. We observed a non-linear decrease of 17% every day. It has stated that cellular growth is possible also with variable flow rate/pressure and it is not affected by our range of values. The differences between theoretical and experimental values were mainly due to the real diameter of the tool used for the fabrication of microchannels, that involves a bigger width of the channels of ca. 2% and, consequentially, a smaller hydraulic resistance and a bigger flowrate. Furthermore, the manufactured microchannels do not have a square shape, but a rectangular shape and the new hydraulic resistance will be equal to 12 *ηL/wa*^3^ [7], where w is the bigger dimension (in this case the width) and a is the smaller dimension (in this case the height). Moreover, critical issues associated with micro-milling manufacturing processes and discussed in Section 2.4 of the main text must be taken into account. Figure S7. Positions of the three regions of interest used for the count of every cell line inside the culture chamber. Regions were selected randomly in order to assess the cell proliferation over the entire surface. This method is helpful in evaluating the homogeneous diffusion of nutrients inside the chamber. Figure S8. Growth of the cells in a specific region of interest. For the HEK293T cell line, a DAPI staining was used. This cell line has a monolayer shape that would have made more difficult the cell counts in absence of fluorescent stain. Figure S9. On the top-left, we can see the growth of the cells during the culture experiment in 48 h. On the bottom-left, we reported the cellular average growth during the experiment and standard deviation evaluated in three different regions of interest. A doubling in the number of cells in 48 h has been observed. On the right there are relevant pictures taken at the end of experiment, (a) BF mode 20X, (b) DIC mode 60X. Figure S10. Growth trend of Melanoma tumor cells in a traditional Petri dish at the same concentration used for the culturing experiment performed in the presented device (ca. 65000/mL). Cells were derived from primary culture and adapted to grow under artificial conditions, i.e., immortalized. Ethical Committees associated with “Istituto Nazionale Tumori IRCCS - Fondazione Pascale” of Naples granted ethical permission. Written informed consent was obtained from all patients in accordance with the Declaration of Helsinki to the use of human biological samples for research purpose.
