# Supplementary material for: A Disposable Passive Microfluidic Device for Cell Culturing
Source: Biosensors (Basel). 2020 Feb 29;10(3):18. doi: 10.3390/bios10030018 (PMC7146476; doi:10.3390/bios10030018)
Supplement: Supplementary file 1 [file biosensors-10-00018-s001.pdf]

biosensors

MDPI

Supplementary: A Disposable Passive Microfluidic Device for Cell Culturing

Table S1. Comparison between the presented device and some of continuous perfusion 2D cell culturing devices in literature.

	Passive	Number of chambers	Area* (mm^2)	Height* (mm)	Flow rate for chamber (µl/h)	Time (days)	Cell lines	Average Percentage Increase (relative to 0 h)**
Presented device	Yes	1	19.6	3	44	2	Melanoma	200%
						3	HeLa, Jurkat, A549, HEK293T	532%, 383%, 250%, 240%
[1]	Yes	96	23.7	15	--	4	C3A	--
[2]	Not	24	35.0	0.2	30	5	SH-SY5Y	200%
[3]	Not	16	7.1	0.5	30	4	Hela, PC12, ASC	--
[4]	Not	280	0.20	0.03	5.85	2	HEK239T	--
[5]	Not	1	290.0	--	--	5	mHSC	--

* Dimensions referred to each singular chamber

** Considering an initial percentage of 100% at 0 h

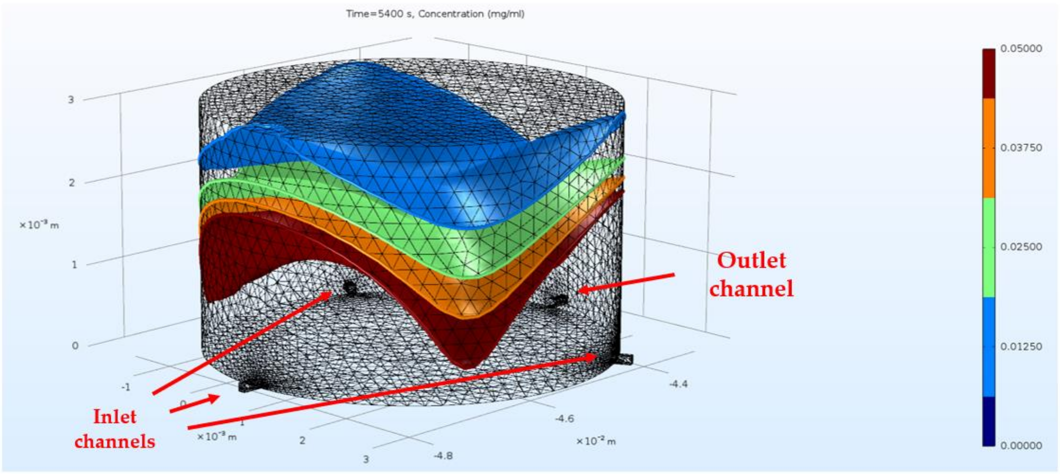

Diffusion coefficient	Initial concentration	Molecular weight	Inlet pressure	Outlet pressure
$1{,}46\,E^{-10}\,\frac{m^2}{s}$	$480\,\frac{ng}{ml}$	$20\,kDa$	$29{,}466\,Pa$	$0\,Pa$

Figure S1. Computational simulation evaluated over 4 hours to assess the faster diffusion of substances in the presented device, comparing with some similar devices in literature [6]. The parameters used for simulation are shown below and are the same used by the authors of the cited article. Unlike their device, having among other things a smaller central culture chamber, a target concentration of 0.025 mg/ml is reached faster (1.5 hour rather than 4 hours) and increases over the time.

Table S2. Constants of interest used during the design of the microfluidic device.

Constants of interest	
**Density of water (ϱ)**	$\mathbf{0.001\ g/mm^3}$
**Viscosity of water (η)**	$\mathbf{0.001\ Pa\cdot s}$
**Gravity acceleration (g)**	$\mathbf{9822\ mm/s^2}$

Thermocouple

We used a K-type thermocouple (Ni-Cr) with fiberglass braid insulation, sold by RS Pro. It is sealed and with exposed probe. Its datasheet is shown in Table S3.

Table S3. Datasheet of Thermocouple RS Pro.

Product	Thermocouple RS Pro
Model	K (Ni-Cr)
Insulation	Fiberglass
Probe diameter	$0.3\ mm$
Operative range	$-50 \div 400°C$
Standards satisfied	RoHS conformity
Response time	$0.7\ s$

Heating Pad

The heating pad used to warm up the environment is produced by SparkFun Electronics. It is able to reach a temperature of $\approx 50°C$, applying a power source of 5V in DC to the cables. It is constructed using a mesh of Polyester filaments and Micro Fiber Conductive Metal, all folded into a protective film of Polyimide. Its datasheet is shown in Table S4, while the warmup profile is shown in Figure S2.

Table S4. Datasheet of the SparkFun Electronics heating pad.

Product	SparkFun Electronics Heating Pad
Operating voltage	$\mathbf{5}\boldsymbol{V}\ \boldsymbol{DC}$
Operating current	$\sim \mathbf{750}\ \boldsymbol{mA}\ (\sim \mathbf{6.5}\ \boldsymbol{\Omega})$
Dimensions	$\mathbf{5} \times \mathbf{10}\ \boldsymbol{cm}$
Standards satisfied	RoHS conformity

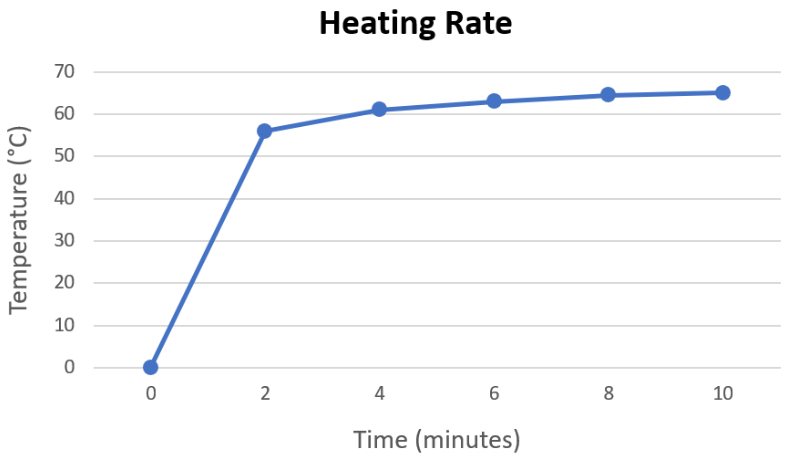

Figure S2. Declared heating rate of the thermic pad used for our experiment and produced by SparkFun Electronics.

Temperature and humidity sensor

There is a crucial part in the experiment to measure and control temperature within the chamber. The temperature has to be set at a desired point of 37°C and it needs to be controlled. Hence, a temperature sensor couplet with a resistance temperature detector (RTD) or with a thermocouple and resistance connected to a 5V power supply will serve the purpose. Humidity should be preferably maintained at 100%, even if in our experiment we reached a range of 70÷80%.

The temperature and humidity sensor used is the DHT22 sold by Arduino and its datasheet is shown in Table S5. The sensor has four pins connected to an Arduino Uno Controller (datasheet shown in Table S6) respectively to:

1. +5V Arduino pin;
2. Arduino pin 2;
3. No connection;
4. GND Arduino pin.

Table S5. Datasheet of DHT22 temperature and humidity sensor.

Product	DHT22
Power supply	$\mathbf{3.3 \div 6}\boldsymbol{V\ DC}$
Output signal	Digital signal via single-bus
Sensing element	Polymer capacitor
Operating range	Humidity $\mathbf{0 \div 100\%}$ RH*<br>Temperature $\mathbf{-40 \div 80°}\boldsymbol{C}$
Accuracy	Humidity $\pm\mathbf{2\%}$ RH (Max $\pm\mathbf{5\%}$ RH)<br>Temperature $< \pm\mathbf{0.5°}\boldsymbol{C}$
Resolution or sensitivity	Humidity $\mathbf{0.1\%}$RH<br>Temperature $\mathbf{0.1°}\boldsymbol{C}$
Repeatability	Humidity $\pm\mathbf{1\%}$ RH<br>Temperature $\pm\mathbf{0.2°}\boldsymbol{C}$
Humidity Hysteresis	$\pm\mathbf{0.3\%}$ RH
Long-term Stability	$\pm\mathbf{0.5\%}$ RH/year
Sensing period Average	$\mathbf{2}\boldsymbol{s}$
Interchangeability	fully interchangeable
Dimensions	small size** $\mathbf{14 \times 18 \times 5.5}$ mm<br>big size $\mathbf{22 \times 28 \times 5}$ mm

* RH stands for Relative Humidity; **Sensor used for our experiments

Table S6. Datasheet of Arduino Uno Controller.

Product	Arduino Uno Controller
Microcontroller	ATmega328
Operating Voltage	$\mathbf{5}\boldsymbol{V}$
Input Voltage (recommended)	$\mathbf{7 \div 12}\boldsymbol{V}$
Input Voltage (limits)	$\mathbf{6 \div 20}\boldsymbol{V}$
Digital I/O Pins	14 (of which 6 provide PWM* output)
Analog Input Pins	$\mathbf{6}$
DC Current per I/O Pin	$\mathbf{40}\ \boldsymbol{mA}$
DC Current for 3.3V Pin	$\mathbf{50}\ \boldsymbol{mA}$
Flash Memory	$\mathbf{32}\ \boldsymbol{KB}$ (ATmega328) of which $\mathbf{0.5}\ \boldsymbol{KB}$ used by bootloader
SRAM	$\mathbf{2}\ \boldsymbol{KB}$ (ATmega328)
EEPROM	$\mathbf{1}\ \boldsymbol{KB}$ (ATmega328)
Clock Speed	$\mathbf{16}\ \boldsymbol{MHz}$

* PWM stands for Pulse-Width Modulation

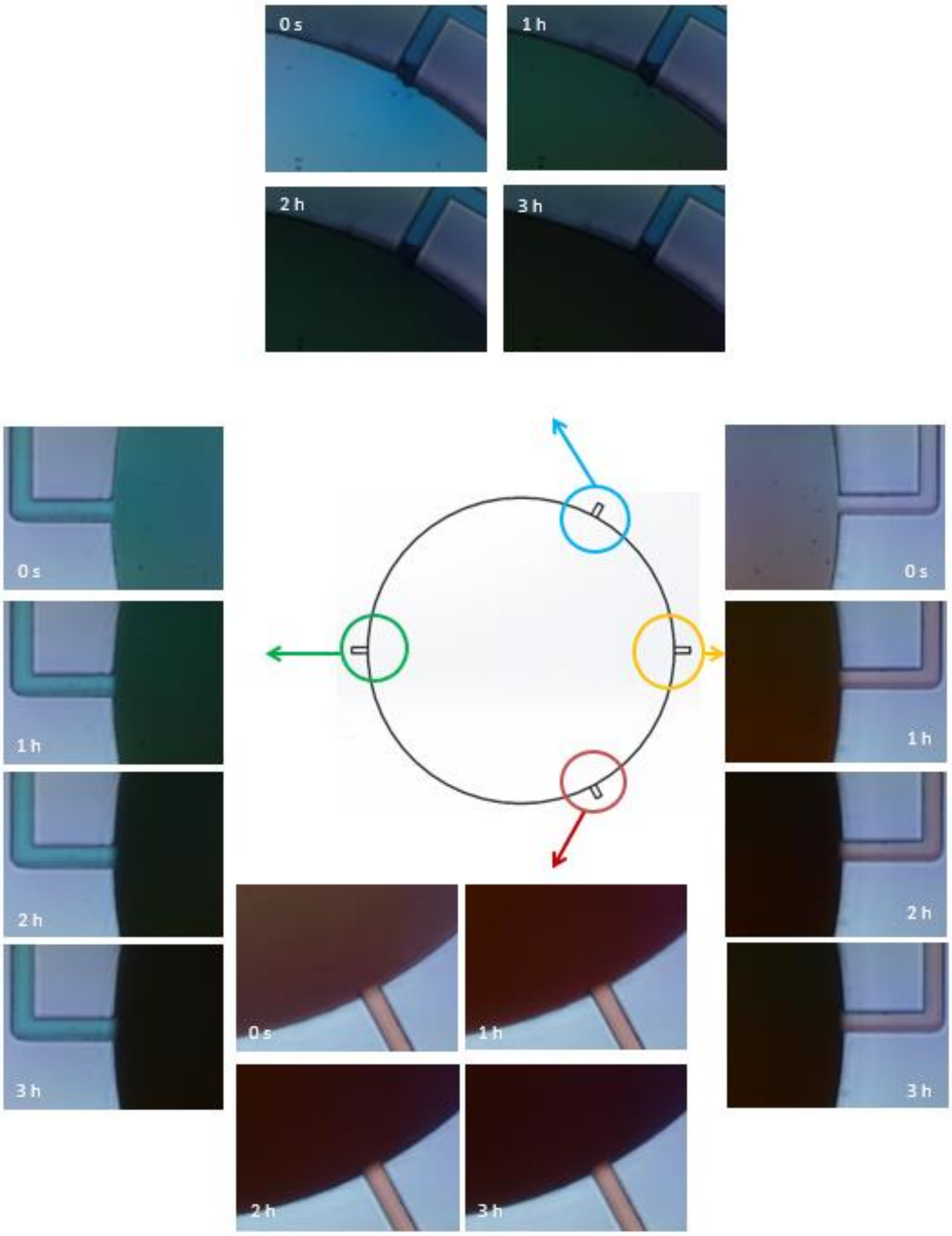
0 s
1 h
2 h
3 h
0 s
1 h
2 h
3 h
0 s
1 h
2 h
3 h
0 s
1 h
2 h
3 h

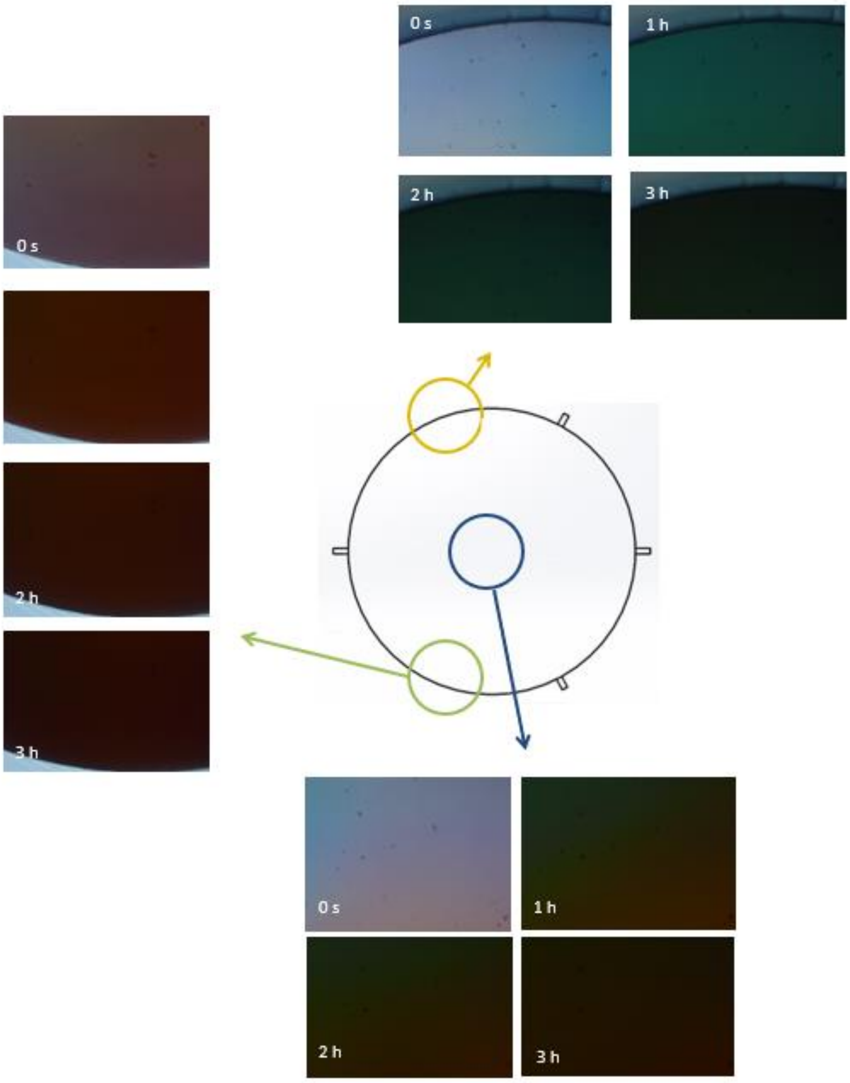

Figure S3. Diffusion of the dyes over time during the experiment with food dyes. The experiment lasted 4 hours, acquiring imaging with the optical inverted microscope in time-lapse from 7 different regions of interest inside the culture chamber.

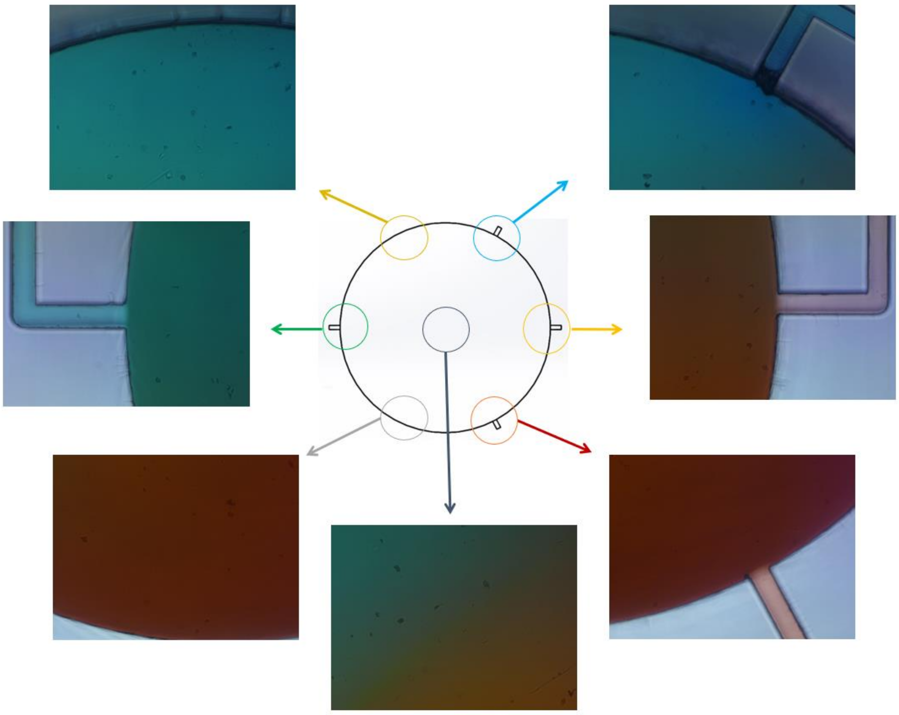

Figure S4. Diffusion of the dyes at ca. 1000 s. We can note how colors are starting to mix with each other, especially in the center of the culture chamber.

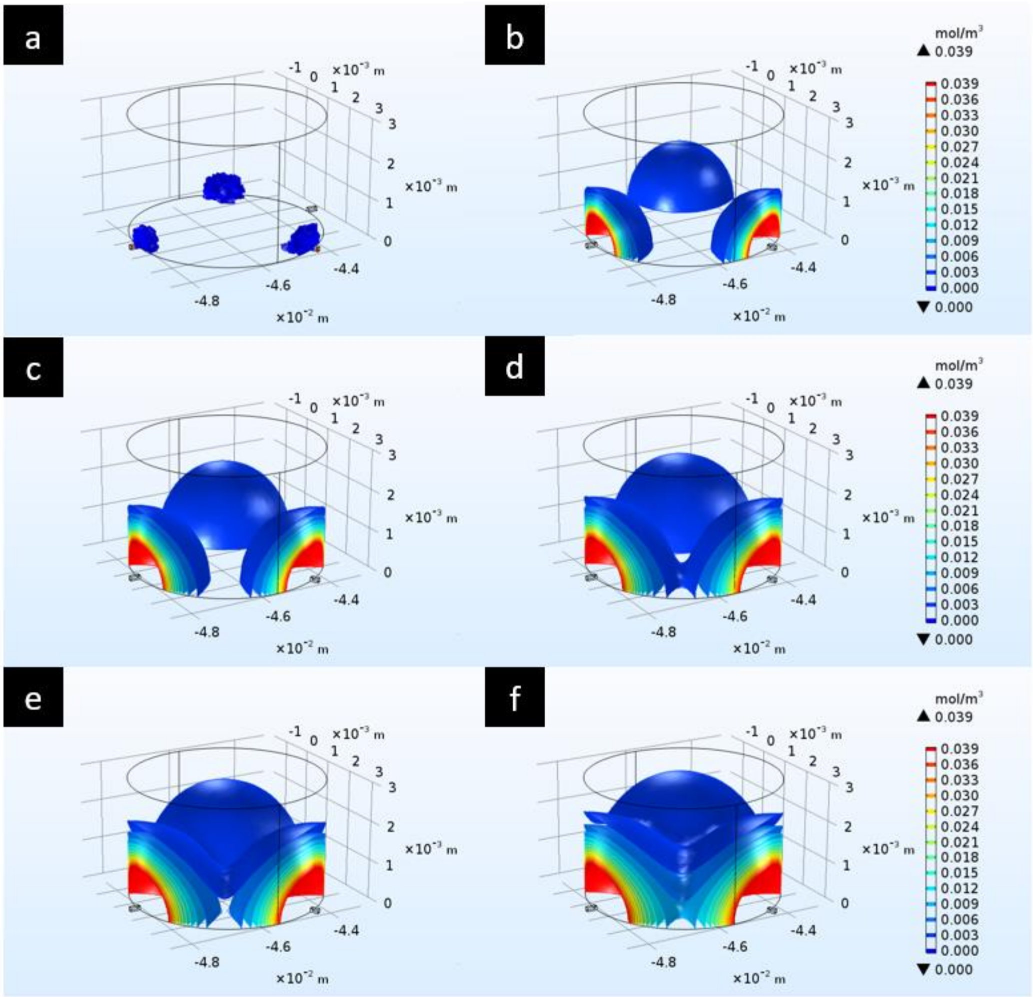

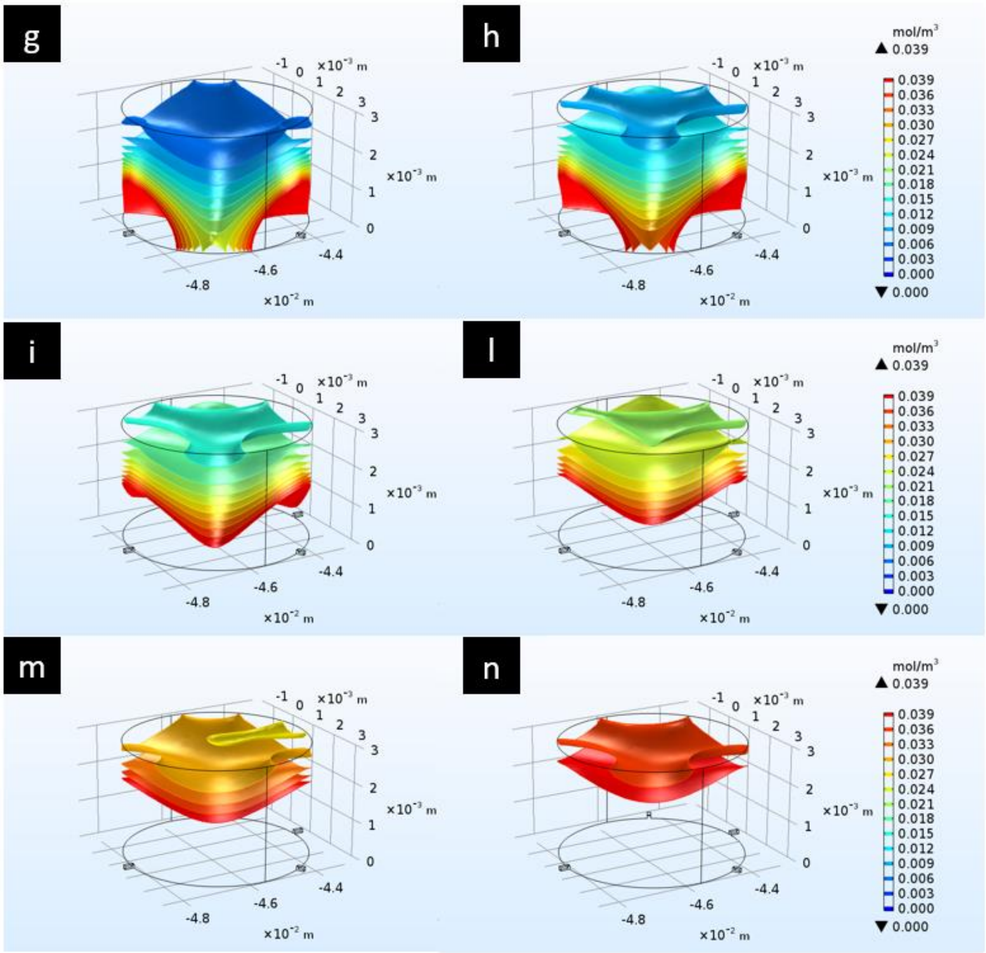

Figure S5. Results obtained from the diffusion simulation of the medium at different time instants [s]: (a) 0, (b) 300, (c) 500, (d) 650, (e) 800, (f) 1000, (g) 1800, (h) 2400, (i) 3000, (l) 3600, (m) 4200, (n) 4800.

Table S7. Comparison between theoretical and experimental measured values of the device.

		**Width (mm)**	**Height (mm)**	**Length (mm)**	**Diameter (mm)**	**Volume (µl)**	**Flow rate (µl/h)**
**Theoretical values**	Total dimensions	80	9	80			
	Inlet channel*	0.1	0.1	60			6.207 (−7%/day)
	Outlet channel	0.1	0.1	20			18.61 (−7%/day)
	Reservoirs		6		20	1884	
	Culture chamber		3		5	58.875	
	Reservoirs total filling volume					5652	
	Predicted total flow rate						18.61 (−7%/day)
**E**	Total dimensions	80	9	80			

Inlet channel*	0.1016	0.1	60			14.9 (−17%/day)
Outlet channel	0.1016	0.1	20			44.7 (−17%/day)
Reservoirs		6		20	1884	
Culture chamber		3		5	59	
Reservoirs total filling volume					5652	
Measured total flow rate						44.7 (−17%/day)

* Values referred to a single inlet channel

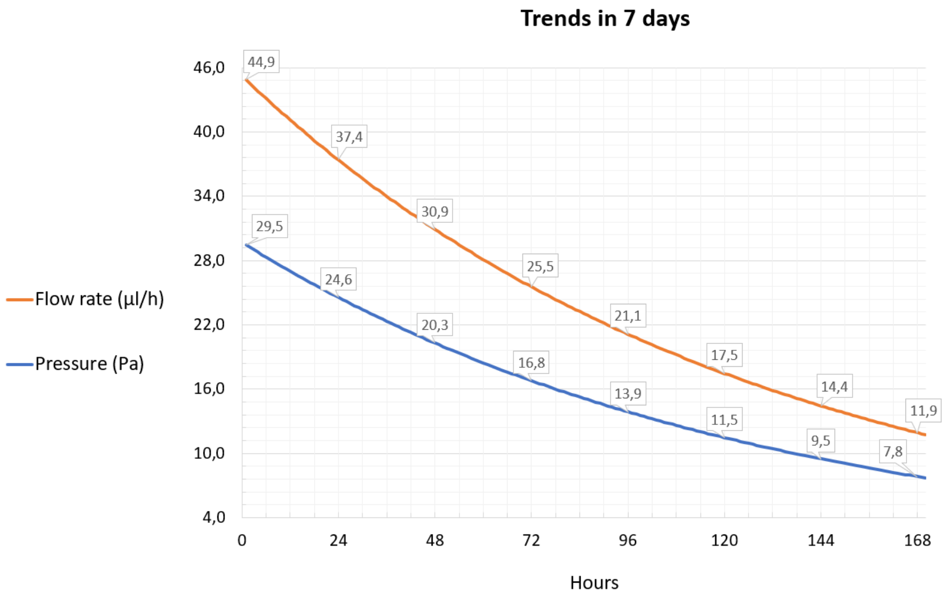

Figure S6. Experimental trends of flow rate and pressure during a week. We observed a non-linear decrease of 17% every day. It has stated that cellular growth is possible also with variable flow rate/pressure and it is not affected by our range of values. The differences between theoretical and experimental values were mainly due to the real diameter of the tool used for the fabrication of microchannels, that involves a bigger width of the channels of ca. 2% and, consequentially, a smaller hydraulic resistance and a bigger flowrate. Furthermore, the manufactured microchannels do not have a square shape, but a rectangular shape and the new hydraulic resistance will be equal to $12\eta L/wa^3$ [7], where w is the bigger dimension (in this case the width) and a is the smaller dimension (in this case the height). Moreover, critical issues associated with micro-milling manufacturing processes and discussed in section 2.4 of the main text must be taken into account.

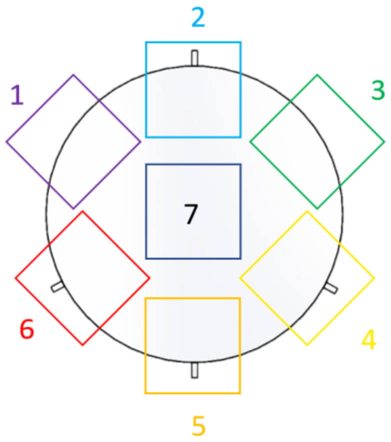

	1	2	3	4	5	6	7
HeLa	✓		✓	✓			
Jurkat		✓	✓				✓
A549		✓				✓	✓
HEK293T	✓		✓		✓		

Figure S7. Positions of the three regions of interest used for the count of every cell line inside the culture chamber. Regions were selected randomly in order to assess the cell proliferation over the entire surface. This method is helpful in evaluating the homogeneous diffusion of nutrients inside the chamber.

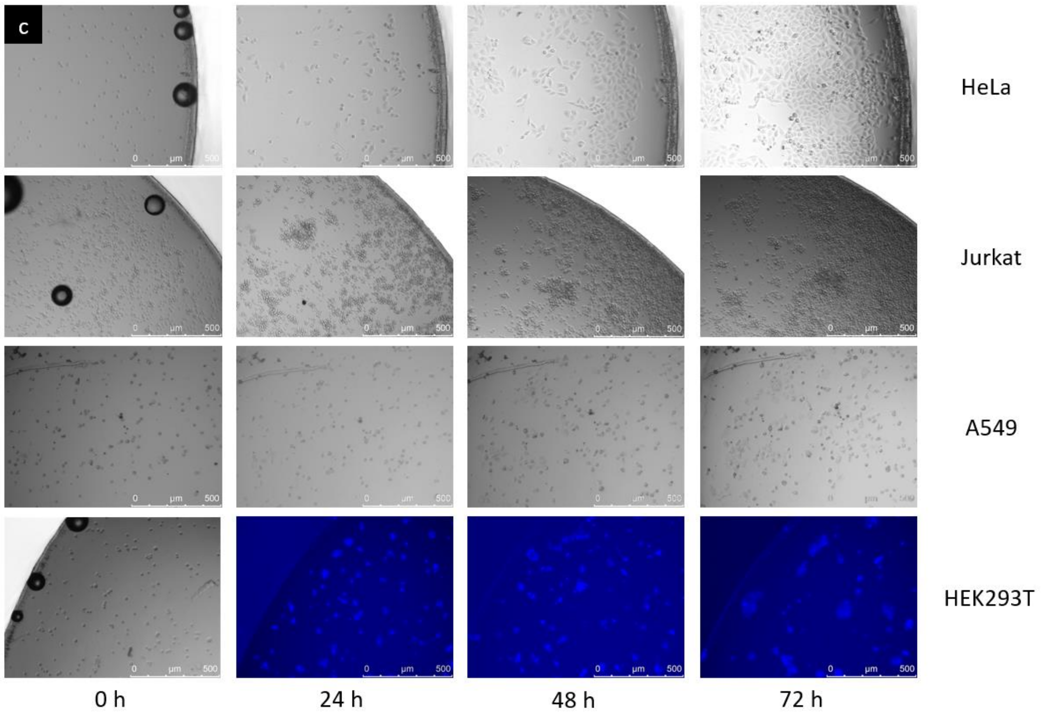

Figure S8. Growth of the cells in a specific region of interest. For the HEK293T cell line, a DAPI staining was used. This cell line has a monolayer shape that would have made more difficult the cell counts in absence of fluorescent stain.

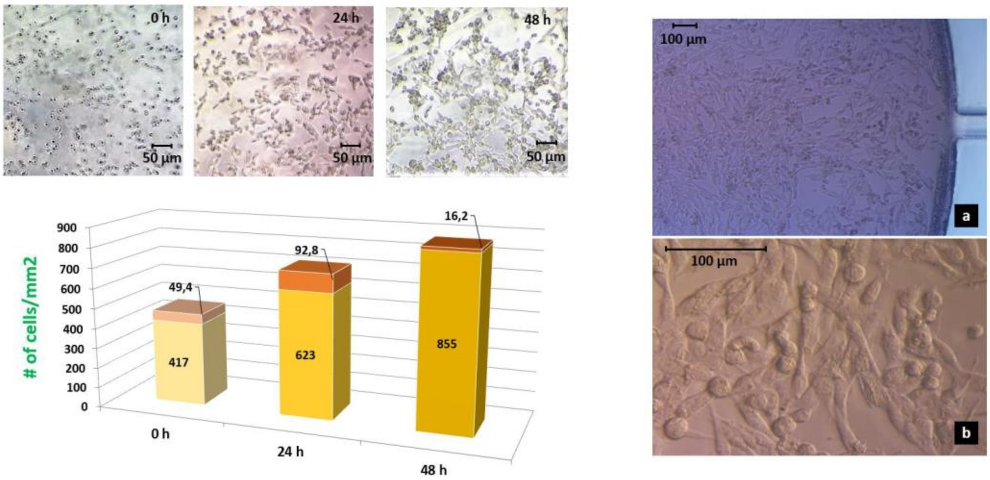

Figure S9. On the top-left, we can see the growth of the cells during the culture experiment in 48 hours. On the bottom-left, we reported the cellular average growth during the experiment and standard deviation evaluated in three different regions of interest. A doubling in the number of cells in 48 hours has been observed. On the right there are relevant pictures taken at the end of experiment, (a) BF mode 20X, (b) DIC mode 60X.

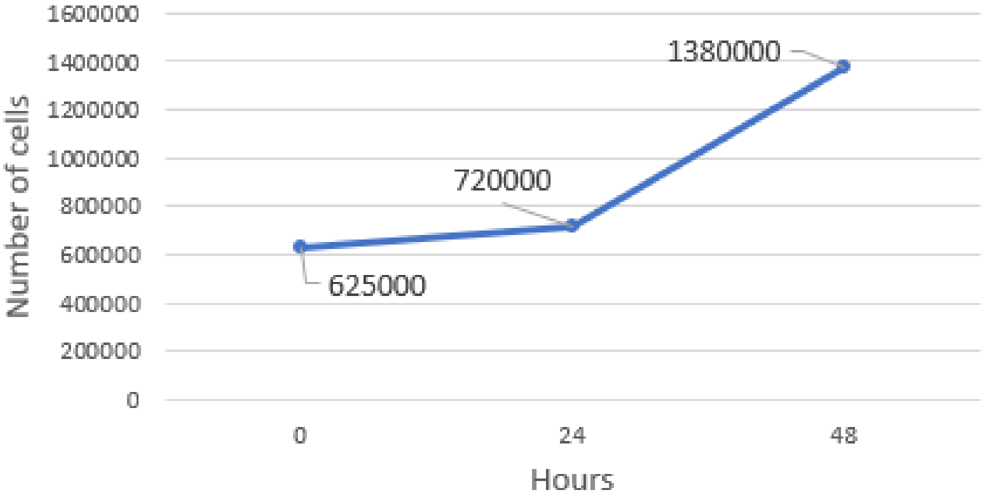

Figure S10. Growth trend of Melanoma tumor cells in a traditional Petri dish at the same concentration used for the culturing experiment performed in the presented device (ca. 65000/ml). Cells were derived from primary culture and adapted to grow under artificial conditions, i.e. immortalized. Ethical Committees associated with "*Istituto Nazionale Tumori IRCCS - Fondazione Pascale*" of Naples granted ethical permission. Written informed consent was obtained from all patients in accordance with the Declaration of Helsinki to the use of human biological samples for research purpose.

Equations

The device can ensure a flow rate ranging from ca. $1\,\mu l/h$ to ca. $18\,\mu l/h$. These values are calculated considering respectively the minimum ($H_{min,\ reservoirs} = 3.1mm$) and the maximum ($H_{max,\ reservoirs} = 6.0mm$) height possible for the fluidic column inside the reservoirs (see Table 1 in the main text for details about technical data of the microfluidic device). If there is no difference on heights between two columns of fluid, there will be no gradient of pressure and, consequentially, no flow rate. It is simple to see in (1), in fact, that when the height of liquid column into reservoirs is

equal to the height of culture chamber (3 mm), there will be a $\Delta H = 0$ and thus a $\Delta P = 0$ and a $\Delta Q = 0$. Pressure, flow rates and resistances calculations are shown below (eq. S1-S4).

$$\Delta P_{max} = \rho \cdot g \cdot \Delta H_{max} = \rho \cdot g \cdot (H_{max,\ reservoirs} - H_{culture\ chamber}) = 29.466\ Pa \quad \text{(S1)}$$

$$R_{inlet\ single\ channel} = 28.454 \frac{\eta \cdot L}{a^4} = 17089.4 \frac{Pa\ \cdot s}{mm^3} \quad \text{(S2)}$$

$$Q_{max,\ inlet\ single\ channel} = \frac{\Delta P_{max}}{R_{inlet}} = 0.001724 \frac{\mu l}{s} = 6.2 \frac{\mu l}{h} \quad \text{(S3)}$$

$$Q_{max,\ total} = Q_{max,\ inlet\ single\ channel} \cdot 3 = 18.6 \frac{\mu l}{h} \quad \text{(S4)}$$

By using the minimum value for the height of reservoirs and following the previous steps, it is possible to find:

$$Q_{min,\ total} = 0.6207 \frac{\mu l}{h} \quad \text{(S5)}$$

The equation that governs the relation between flowrate and height is the following:

$$Q = \frac{P}{R} = \frac{\rho \cdot g \cdot H}{R} \rightarrow \Delta Q = \frac{\rho \cdot g \cdot \Delta H}{R} \quad \text{(S6)}$$

It has been demonstrated previously that with H_{max} it is possible to generate a flow rate of 18.6 $\mu l/h$. The volume inside a cylindrical reservoir of radius r, base surface S and height H is equal to:

$$V = S \cdot H = \pi \cdot r^2 \cdot H \quad \text{(S7)}$$

We calculated the percentage variation of the height of the fluidic column (and hence also of the flowrate and pressure) by implementing an iterative calculation on a spreadsheet (Excel) and by assuming that the flowrate is maintained constant for 1 hour every time. With these conditions, we made the following calculation above. The total lost volume in 1 hour from the three reservoirs is equal to:

$$V_{lost,1h} = Q_{max} \cdot 1\text{h} = 18.6\ \mu\text{l} = 18.6\ mm^3 \quad \text{(S8)}$$

The height lost from every single reservoir is equal to:

$$\Delta H_{1h} = \frac{V_{lost,1h}}{S_{total}} = \frac{V_{lost,1h}}{S \cdot 3} = \frac{18.6\ mm^3}{942\ mm^2} = 0.0197\ \text{m}m \quad \text{(S9)}$$

The new value of height will be equal to:

$$H_{final} = H_{initial} - \Delta H_{1h} = H_{max,\ reservoirs} - \Delta H_{1h} = (6 - 0.0197)mm = 5.9803\ mm \quad \text{(S10)}$$

This means that, the percentage variation per hour of height (hence of pressure and flow rate) is:

$$\%\ variation = \frac{H_{final} - H_{initial}}{H_{initial}} = \frac{(5.9803 - 6)\ mm}{6\ mm} = -0.33\%/\text{h} \quad \text{(S11)}$$

By repeating these calculations with the new values of pressure, height and flowrate, it is possible to find a decrease of ca. 7% for every operative day.

References

1. Goral, V.N.; Zhou, C.; Lai, F.; Yuen, P.K. A continuous perfusion microplate for cell culture. *Lab Chip* **2013**, *13*, 1039-1043.
2. Li, R.; Lv, X.; Hasan, M.; Xu, J.; Xu, Y.; Zhang, X.; Qin, K.; Wang, J.; Zhou, D.; Deng, Y. A Rapidly Fabricated Microfluidic Chip for Cell Culture. *Journal of Chromatographic Science* **2015**, *54*, 523-530.

3. Sabourin, D.; Skafte-Pedersen, P.; Soe, M.J.; Hemmingsen, M.; Alberti, M.; Coman, V.; Petersen, J.; Emneus, J.; Kutter, J.P.; Snakenborg, D., et al. The MainSTREAM component platform: a holistic approach to microfluidic system design. *J Lab Autom* **2013**, *18*, 212-228.
4. Woodruff, K.; Maerkl, S.J. A High-Throughput Microfluidic Platform for Mammalian Cell Transfection and Culturing. *Scientific reports* **2016**, *6*, 23937.
5. Jaccard, N.; Macown, R.J.; Super, A.; Griffin, L.D.; Veraitch, F.S.; Szita, N. Automated and online characterization of adherent cell culture growth in a microfabricated bioreactor. *J Lab Autom* **2014**, *19*, 437-443.
6. Moore, T.A.; Young, E.W. Single cell functional analysis of multiple myeloma cell populations correlates with diffusion profiles in static microfluidic coculture systems. *Biomicrofluidics* **2016**, *10*, 044105.
7. Perozziello, G.; Simone, G.; Geschke, O.; Di Fabrizio, E. Theory. In *Microfluidic System Interfacing - An onverview on packaging of microfluidic systems, how to interface them fluidically, electrically and optically*, 1st ed.; LAP LAMBERT Academic Publishing GmbH & Co. KG: Saarbrücken, Germany, 2011; pp. 16-19.
